# MRI findings in six dogs with ependymoma of the brain and spinal cord

**DOI:** 10.1111/vru.13477

**Published:** 2024-12-16

**Authors:** John F Griffin, William S Stevenson, Nathan C Nelson, Annie V Chen, Silke Hecht, Brian F Porter, C Elizabeth Boudreau, Swan Specchi, Marco Bernardini, Wilfried Mai

**Affiliations:** ^1^ Department of Large Animal Clinical Sciences Texas A&M University College Station Texas USA; ^2^ Columbia Veterinary Emergency Trauma and Specialty Columbia South Carolina USA; ^3^ Department of Molecular Biomedical Sciences College of Veterinary Medicine, North Carolina State University Raleigh North Carolina USA; ^4^ Veterinary Referral Center of Central Oregon Bend Oregon USA; ^5^ Department of Small Animal Clinical Sciences College of Veterinary Medicine, University of Tennessee Knoxville Tennessee USA; ^6^ Department of Veterinary Pathobiology Texas A&M University College Station Texas USA; ^7^ Department of Small Animal Clinical Sciences Texas A&M University College Station Texas USA; ^8^ Diagnostic Imaging Department AniCura Ospedale Veterinario “I Portoni Rossi” Bologna Italy; ^9^ Antech Imaging Services Fountain Valley California USA; ^10^ Department of Animal Medicine Production and Health, University of Padua Legnaro Italy; ^11^ Department of Clinical Sciences and Advanced Medicine University of Pennsylvania School of Veterinary Medicine, Section of Radiology Philadelphia Pennsylvania USA

**Keywords:** central nervous system, CNS, magnetic resonance imaging, neoplasia, tumor

## Abstract

There are few published descriptions of the MRI appearance of canine intracranial or spinal cord ependymoma. In this multicenter, retrospective, secondary analysis, case series study, three veterinary radiologists independently reviewed and recorded imaging characteristics of MRI studies in six dogs with histopathologically confirmed ependymoma (three intracranial and three spinal cord cases). A consensus was reached when there was disagreement on specific features. All intracranial ependymomas had forebrain location, heterogeneous signal intensity in T1‐weighted (T1W) and T2‐weighted (T2W) images, heterogeneous contrast enhancement, and hyperintensity in T2W images. Two ependymomas had an intraventricular location; one was intra‐axial. Other imaging features included intralesional cyst‐like structures, intralesional hemorrhage, and perilesional edema. Dogs with spinal cord ependymoma had intramedullary lesions located in the cervical or thoracic spinal cord. Spinal cord ependymomas were isointense and homogeneous in T1W images and hyperintense in T2W images. Lesion location relative to the central canal of the spinal cord was variable. All three spinal cord ependymomas had perilesional T2W hyperintensity and moderate, heterogeneous contrast enhancement. None of the spinal cord ependymomas had intralesional cyst‐like structures. One spinal cord ependymoma had evidence of drop metastases (diffuse, leptomeningeal). MRI features of canine ependymomas overlap with those of other diseases of the brain and spinal cord. Ependymoma should be considered a differential diagnosis for dogs with intraventricular, intra‐axial forebrain, or intramedullary spinal cord masses.

## 1 INTRODUCTION

Canine ependymomas are uncommon neoplasms that arise from the ependyma, the ciliated epithelial cell lining of the ventricular system and central canal of the spinal cord.^1^, ^2^ Canine intracranial ependymomas are most commonly located within or adjacent to the lateral and third ventricles.^3^ They have been reported to have well‐defined margins, low or intermediate T1‐weighted (T1W) signal intensity, high T2‐weighted (T2W) signal intensity, variable contrast enhancement, minimal peritumoral edema, and occasional intralesional cyst‐like structures and hemorrhage.^2^, ^4^ Other differential diagnoses for ventricular‐associated tumors in dogs include choroid plexus tumors (CPTs), ventricular meningiomas, oligodendrogliomas, lymphoma, and neurocytomas.^4^, ^5^, ^6^, ^7^, ^8^, ^9^, ^10^, ^11^, ^12^

Canine spinal cord ependymomas are often located in the thoracic or lumbar spinal cord and can cause a focal mass or have a multisegmental, fusiform pattern.^13^ They have similar MRI signal characteristics to their intracranial counterparts.^13^ Other differential diagnoses for intramedullary spinal cord lesions in dogs include astrocytoma, oligodendroglioma, metastatic neoplasia, lymphoma, histiocytic sarcoma, meningomyelitis, ischemic myelopathy (including fibrocartilaginous embolism), and contusion.^14^

Published MRI findings in canine ependymoma are sparse. The existing literature includes a few case reports, examples in textbooks, and briefly described examples appearing in larger case series of dogs with multiple types of neoplasia.^4^, ^6^, ^7^, ^10^, ^11^, ^13^, ^15^, ^16^, ^17^ Therefore, this paper aims to provide a detailed description of the MRI findings in a larger population of dogs with ependymoma of the brain and spinal cord.

## 2 MATERIAL AND METHODS

This was a multicenter, retrospective, secondary analysis, case series design. Medical records at Texas A&M University (TAMU), North Carolina State University (NCSU), Washington State University (WSU), and I Portoni Rossi Veterinary Hospital (OVPR) were searched for client‐owned dogs with histopathologically confirmed ependymoma of the brain or spinal cord and MRI of the lesion (consecutive enrollment). To be included, cases were required to have at least T1W images before and after administration of gadolinium‐based contrast medium and T2W images. If additional sequences were available, they were also interpreted. When histopathologic slides were available, they were reviewed by a board‐certified pathologist to confirm the diagnosis. As this was a retrospective study, institutional animal care and use committee approval was not needed. Each hospital director approved the use of hospital data for this study.

Clinical data were extracted from the medical records by a licensed veterinarian (W.S.S.). The following data were recorded: contributing institution, age, sex, breed, body weight, clinical signs, neuroanatomical localization, duration and progression of clinical signs, date of MRI, date of tissue sampling for histopathology, and cause of death. When available, treatments administered were recorded. The DICOM Images were evaluated by three board‐certified radiologists (S.S., W.M., and S.H.) experienced in the interpretation of MRI of the brain and spinal cord. Evaluators were aware of the final diagnosis. Each evaluator reviewed the images using the DICOM viewer of their choice. Images were evaluated independently, and MRI findings were recorded using standardized image analysis forms (see Table 1). Briefly, evaluators were asked to record the number, location, signal characteristics, margins, evidence of a mass effect, and perilesional changes. Spinal cord lesion location in relationship to the central canal was classified (as centered on the central canal, not centered on the central canal, or involving the entire cross‐sectional area of the spinal cord) in an attempt to identify an imaging feature that might differentiate lesions originating from the central canal from those originating from the neuroparenchyma. After all images were assessed independently, instances of discrepancy were discussed by video conference, and a consensus rating was agreed upon to report. Evaluator responses were used to generate standardized MRI descriptions and tabulated to identify consistent findings within our population.

**TABLE 1:** Image analysis forms for intracranial and spinal cord ependymomas.

Form
Both How many lesions are present? 1, 2, 3, ≥4
IC: How does the lesion relate to the ventricles and subarachnoid space? Intraventricular, periventricular, no ventricular contact (intra‐axial), no ventricular contact (extra‐axial)
IC: If the lesion is ventricular‐associated, which ventricle? Right lateral, left lateral, third, fourth
IC: With which brain division is the lesion center most closely associated with? Right/left, telencephalon, diencephalon, mesencephalon, pons, cerebellum, medulla oblongata
S: Which spinal cord segments are involved? C1‐C5, C6‐T2, T3‐L3, L4‐S2, S3‐Cd
S: Which compartment is the lesion centered on? IM, IDEM, ED
S: Does the lesion secondarily involve other compartments? IM, IDEM, ED
S: How does the IM lesion relate to the CC? Centered on the CC, not centered on the CC, involves entire spinal cord cross‐sectional area
Both: Are there intralesional cyst‐like structure(s)? Yes, no
Both: Do the intralesional cyst‐like structure(s) suppress on T2W‐FLAIR? Yes, no, not available
Both: Is there evidence of perilesional edema (T2‐hyperintensity)? Yes, no
Both: On T1W (precontrast) images, describe the predominate SI relative to NAGM. High, iso, low
Both: On T1W images (precontrast), characterize lesion SI uniformity. homogeneous, heterogenous
Both: Describe lesion contrast enhancement. Yes—mild, yes—moderate, yes—strong, none
Both: If contrast enhancement is present, characterize its distribution. Homogeneous, heterogeneous (with or without peripheral enhancement)
Both: If contrast enhancement is present, describe the margins of the contrast‐enhancing portion of the lesion. Well‐circumscribed, not well‐circumscribed, variable
Both: On T2W images, describe the predominate lesion SI relative to NAGM. High, iso, low
Both: On T2W images, characterize lesion SI uniformity. Homogeneous, heterogeneous
Both: Describe margins of lesion in T2W images. Well‐circumscribed, not well‐circumscribed, variable
Both: On T2*W images, if available, grade the presence of hypointensity. Present—with peripheral hypointensity, present—without peripheral hypointensity, absent, not available
IC: Is there evidence of a midline shift? Absent, present toward right, present toward left
IC: Is there evidence of subfalcine herniation? Absent, present toward right, present toward left
IC: Is there evidence of transtentorial herniation? Present ‐ rostral, present ‐ caudal, or absent.
IC: Is there evidence of cerebellar herniation through foramen magnum? Present, absent
S: Is there evidence of syringohydromyelia? Yes—cranial to lesion, yes—caudal to lesion, yes—cranial and caudal to lesion, no

Abbreviations: C1, first cervical spinal cord segment; C5, fifth cervical spinal cord segment; C6, sixth cervical spinal cord segment; CC, central canal; Cd, caudal spinal cord segments; ED, extradural; IC, intracranial; IDEM, intradural‐extramedullary; IM, intramedullary; iso, isointense; L3, third lumbar spinal cord segment; L4, fourth lumbar spinal cord segment; NAGM, normal‐appearing gray matter; NAGM, normal‐appearing gray matter; S, spinal; S2, second sacral spinal cord segment; S3, third sacral spinal cord segment; SI, signal intensity; SI, signal intensity; T1W, T1‐weighted; T1W, T1‐weighted; T2*W, T2*‐weighted.; T2, second thoracic spinal cord segment; T2W, T2‐weighted; T2W, T2‐weighted, FLAIR, fluid‐attenuated inversion recovery; T3, third thoracic spinal cord segment.

## 3 RESULTS

The initial medical records search yielded nine potential cases to include (five intracranial and four spinal cord). Slides from TAMU and OVPR were available for secondary histopathology review. Slides from NCSU and WSU were unavailable for secondary histopathology review. Three cases were excluded because of inconclusive secondary histopathology reviews (two intracranial and one spinal cord). Hence, six dogs were ultimately included (three intracranial, three spinal cord). Two of the spinal cord cases had been included in a prior study that did not aim specifically at describing MRI characteristics.^14^ The intracranial cases were imaged at 3 tesla, 1.5 tesla, and 0.22 tesla. Two spinal cord cases were imaged at 1 tesla and one at 0.22 tesla. The MRI acquisition parameters are included in Tabels S1 and S2. In addition to T2W images and T1W images made before and after contrast medium administration, all intracranial dogs had T2W fluid‐attenuated inversion recovery (FLAIR) images, and two intracranial dogs had T2*‐weighted images (T2*W).

All dogs with intracranial ependymoma had forebrain lesions, heterogeneous signal intensity in T1W and T2W images, heterogeneous contrast enhancement without peripheral enhancement, hyperintensity to normal‐appearing gray matter (NAGM) in T2W images, and caudal transtentorial herniation. The following features were found in two out of three dogs: intraventricular location, intralesional cyst‐like structures that suppressed in T2W FLAIR images, perilesional edema, isointensity to NAGM in T1W images, strong contrast enhancement with well‐defined margins, poorly circumscribed margins in T2W images, and midline shift away from the lesion. None of the dogs had brain herniation through the foramen magnum.

**FIGURE 1:**
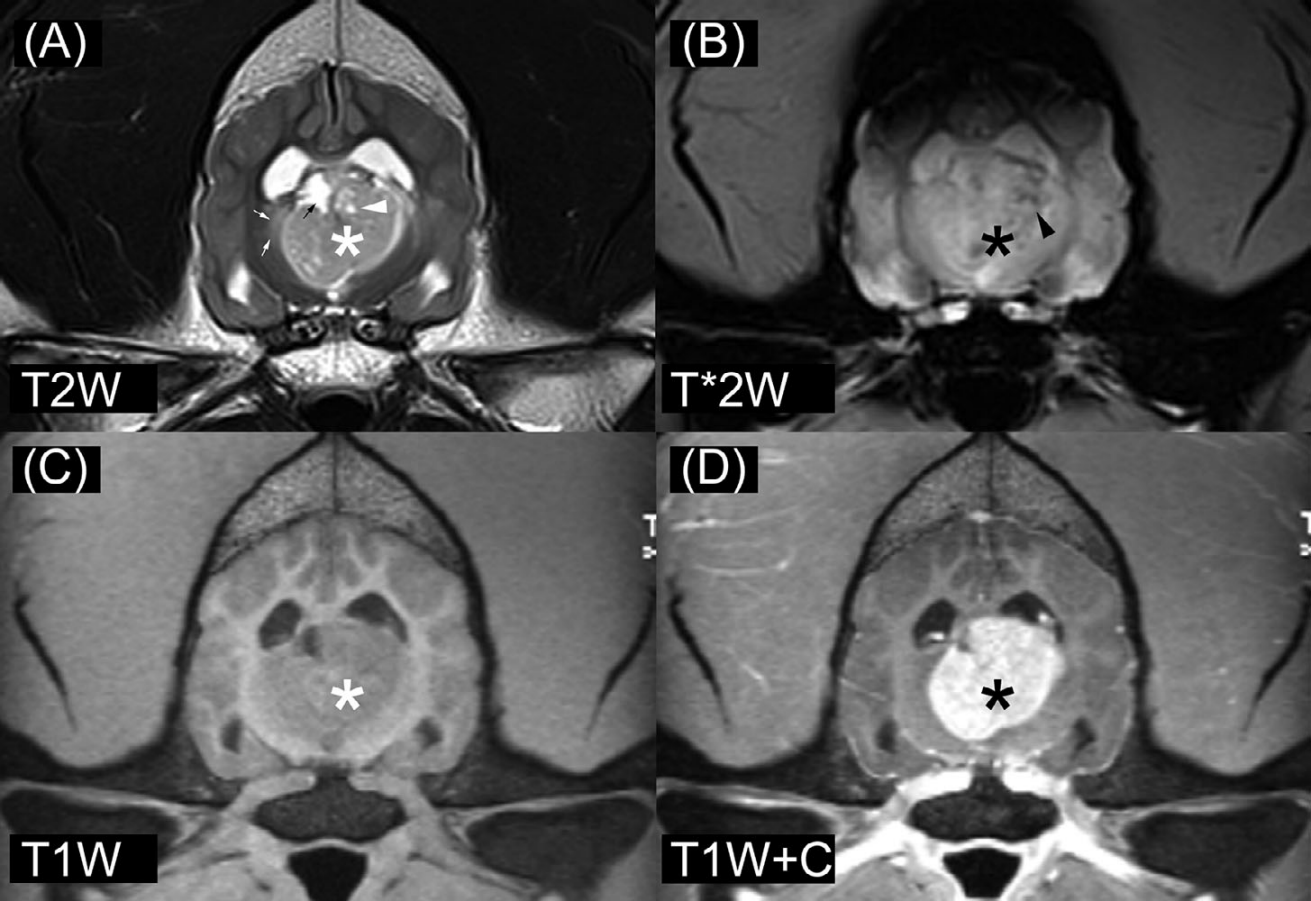
MRI of ependymoma in a 5‐year‐old Rottweiler (field strength = 3 tesla). There is a contrast‐enhancing mass involving the third ventricle and thalamus (*). A, The white arrowhead denotes a small area of intralesional fluid accumulation. The small white arrows denote the T2W hyperintensity of the adjacent brain (presumed vasogenic edema). The small black arrow denotes the third ventricle, which is displaced and abnormally shaped. B, The black arrowhead denotes a small region of reduced signal intensity, thought to represent intralesional hemorrhage. B, There is a susceptibility artifact involving the dorsal aspect of the brain. This is attributed to the close proximity of the air‐filled frontal sinuses. T2W, T2‐weighted image; T2*W, T2*‐weighted image; T1W, T1‐weighted image; T1W+C, T1‐weighted image made after gadolinium‐based contrast medium administration.

The first intracranial ependymoma dog (Figure 1) was a 5‐year‐old, 40.5 kg, male Rottweiler (from TAMU). He had progressive clinical signs of a 10‐day duration. He was lethargic, mentally dull, and had polyuria/polydipsia. He was apparently blind and would run into objects. On MRI, he had a single lesion in the third ventricle, most closely associated with the left diencephalon. There were intralesional cyst‐like structures that were suppressed in T2W FLAIR images. The lesion was isointense to NAGM in T1W images and hyperintense to NAGM in T2W images with perilesional T2‐hyperintensity. Lesion signal intensity was heterogeneous in both T1W and T2W images. Contrast enhancement was strong and heterogeneous without peripheral enhancement. Lesion margins were well‐circumscribed in both T1W postcontrast and T2W images. T2*W images showed hypointensity or blooming artifacts. The distribution of the hypointensity was not peripheral. The dog had a midline shift to the right without subfalcine brain herniation. He was humanely euthanized on the same day as the MRI, and a necropsy was subsequently performed.

**FIGURE 2:**
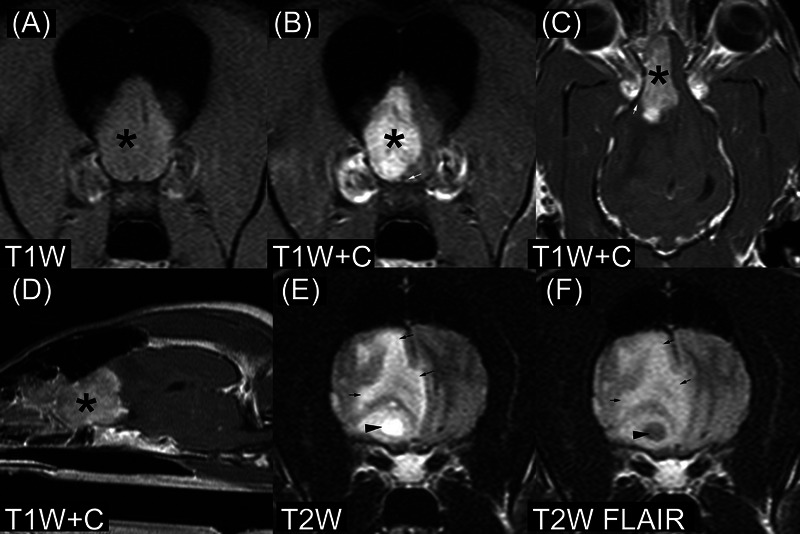
MRI of ependymoma in an 11‐year‐old German shepherd dog (field strength = 1.5 tesla). A and B are located just rostral to the optic canals. E and F are T2‐weighted (T2W) and T2W‐fluid attenuated inversion recovery (FLAIR) images located just rostral to the optic chiasm. A–D, There is a contrast‐enhancing intra‐axial mass involving the right olfactory region (*). E, F, There is an intralesional cyst‐like structure that mostly suppresses on the T2W FLAIR images (black arrowhead). The small white arrows in B and C denote an acute angle formed at the pial surface between the mass and the adjacent brain. This is an example of the “claw sign”, which suggests intra‐axial location.^37^ The small black arrows in E and F denote presumed vasogenic edema. The dog also has a leftward midline shift and subfalcine herniation. T1W, T1‐weighted image; T1W+C, T1‐weighted image made after gadolinium‐based contrast medium administration; T2W, T2‐weighted image; T2W FLAIR, T2‐weighted fluid‐attenuated inversion recovery.

The second intracranial ependymoma dog (Figure 2) was an 11‐year‐old, 27 kg, male castrated German shepherd dog (from NCSU). He had progressive clinical signs of 21‐day duration, beginning with hyporexia and lethargy and progressing to pacing, circling to the right, staring into space, and neck pain. On MRI, he had a single intra‐axial lesion of the right telencephalon with no ventricular contact. There were intralesional cyst‐like structures that were suppressed in T2W FLAIR images. The lesion was hypointense to NAGM in T1W images and hyperintense to NAGM in T2W images with perilesional T2‐hyperintensity. Signal intensity was heterogeneous in both T1W and T2W images. Contrast enhancement was strong and heterogeneous without peripheral enhancement. Lesion margins were well‐circumscribed in T1W postcontrast images and not well‐circumscribed in T2W images. T2*W images were available and negative for hypointensity and blooming artifacts. The dog had a midline shift to the left and subfalcine herniation. He was treated with hydroxyurea (18 mg/kg by mouth every 24 h) and prednisone (0.3 mg/kg by mouth every 12 h). He was humanely euthanized 38 days after the MRI and necropsy was performed.

**FIGURE 3:**
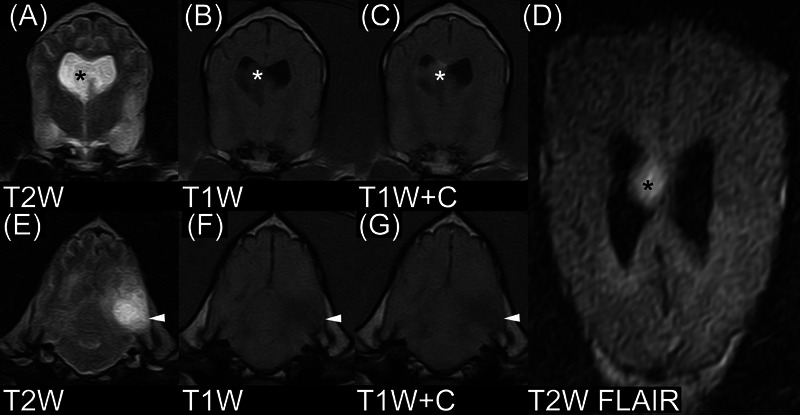
MRI of ependymoma in a 10‐year‐old boxer (field strength = 0.22 tesla). A–C, The asterisks denote a mass in the right lateral ventricle. The mass silhouettes with the CSF in (A) and has mild contrast enhancement in (C). It is easier to see in (D) compared with (A) because the cerebrospinal fluid has been attenuated. E–G, There is a noncontrast enhancing intra‐axial mass in the left occipital lobe. Histopathology was not available for this mass, and it was thought to most likely represent a glioma or, less likely, a postictal change. T2W, T2‐weighted image; T1W, T1‐weighted image; T1W+C, T1‐weighted image made after gadolinium‐based contrast medium administration; T2W FLAIR, T2‐weighted fluid‐attenuated inversion recovery.

The third intracranial ependymoma dog (Figure 3) was a 10‐year‐old, 35 kg female boxer from OVPR). She had progressive clinical signs of a 21‐day duration. Her clinical signs included barking at night, restlessness, aggression, and tonic‐clonic seizures. On MRI, she had an intraventricular lesion of the right lateral ventricle, most closely associated with the right telencephalon. In addition, there was a large intra‐axial mass in the left occipital lobe. As it relates to the intraventricular mass, there were no intralesional cyst‐like structures. The lesion was isointense to NAGM in T1W images and hyperintense to NAGM in T2W images without perilesional T2‐hyperintensity. Signal intensity was heterogeneous in both T1W and T2W images. Contrast enhancement was mild and heterogeneous without peripheral enhancement. Lesion margins were variable in T1W postcontrast images and not well‐circumscribed in T2W images. T2*W images were not available. The dog had no midline shift. The dog was humanely euthanized on the same day as the MRI, and samples of the intraventricular mass were submitted for histopathology. Samples of the intra‐axial left occipital lobe mass were not obtained.

All 3 cases of spinal cord ependymoma had intramedullary lesions without involvement of the intra‐dural‐extramedullary or extradural compartment. None of these dogs had T2W‐FLAIR or T2*W images available. Spinal cord ependymomas were all isointense to NAGM in T1W images and hyperintense to NAGM in T2W images, had homogeneous signal intensity in T1W images, had perilesional T2‐hyperintensity, and had moderate contrast enhancement that was heterogeneous without peripheral enhancement. None of these cases had intralesional cyst‐like structures.

**FIGURE 4:**
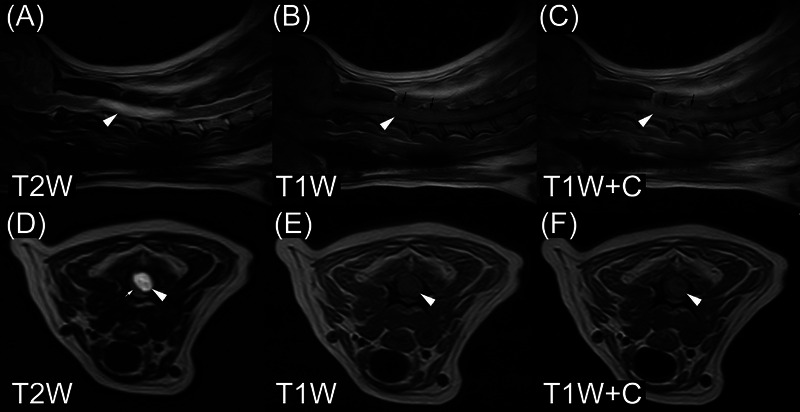
MRI of ependymoma in a 7‐year‐old Yorkshire terrier (field strength = 0.22 tesla). D–F are located at the cranial aspect of the third cervical vertebrae. The white arrowheads denote a contrast‐enhancing, expansile, intramedullary mass extending from the second through fifth cervical vertebrae. The small white arrow in (D) denotes the normal appearance of the spinal cord surrounding the mass. The mass is not centered on the central canal of the spinal cord. The small black arrows in (B) and (C) denote the thinning of the cortex of the laminae of the third and fourth cervical vertebrae, thought to indicate pressure atrophy secondary to the spinal cord mass. Note the mild vertebral canal enlargement. T2W, T2‐weighted image; T1W, T1‐weighted image; T1W+C, T1‐weighted image made after gadolinium‐based contrast medium administration.

The first spinal cord ependymoma dog (Figure 4) was a 7‐year‐old, 5 kg, male Yorkshire terrier (from OVPR). He had progressive clinical signs of a 40‐day duration. His clinical signs included depression, general proprioceptive ataxia, tetraparesis, and neck pain. On MRI, he had an intramedullary lesion involving the third through fifth cervical spinal cord segments. The lesion was not centered on the central canal of the spinal cord. Signal intensity was homogeneous in T2W images. Lesion margins were well‐circumscribed in both T1W postcontrast and T2W images. The dog did not have syringohydromyelia and was euthanized on the same day as the MRI. Samples of the mass were collected for histopathology.

**FIGURE 5:**
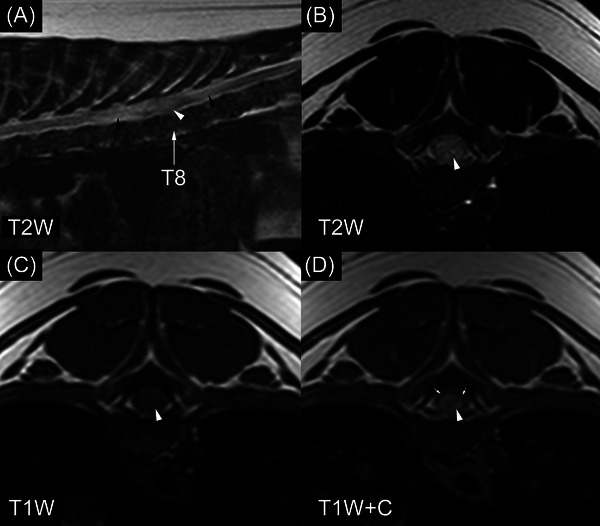
MRI of ependymoma in a 1‐year‐old dachshund (field strength = 1 tesla). B–D, are located at the cranial aspect of the eighth thoracic vertebra. There is a contrast‐enhancing, expansile, intramedullary mass, which is centered on the central canal of the spinal cord (white arrowheads). The black arrowheads in (A) denote spinal cord T2‐hyperintensity cranial and caudal to the mass. This was caused by a combination of syringohydromyelia and spinal cord hyperintensity (presumed edema). The small white arrows in (D) denote a thin rim of normal‐appearing spinal cord surrounding the contrast‐enhancing mass. This part of the spinal cord is indistinguishable from the mass in (B). T8, eighth thoracic vertebra, T2W, T2‐weighted image; T1W, T1‐weighted image; T1W+C, T1‐weighted image made after gadolinium‐based contrast medium administration.

The second spinal cord ependymoma dog (Figure 5) was a 1‐year‐old, 4 kg, female spayed dachshund (from WSU). She had a seven‐day history of progressive paraparesis progressing to paraplegia. On MRI, she had an intramedullary lesion involving the eighth thoracic spinal cord segment. The lesion was centered on the central canal of the spinal cord. Signal intensity was heterogeneous in T2W images. Lesion margins were well‐circumscribed in T1W postcontrast and not well‐circumscribed in T2W images. The dog had syringohydromyelia cranial and caudal to the lesion. A biopsy was performed nine days after the MRI. Treatments administered were not available.

**FIGURE 6:**
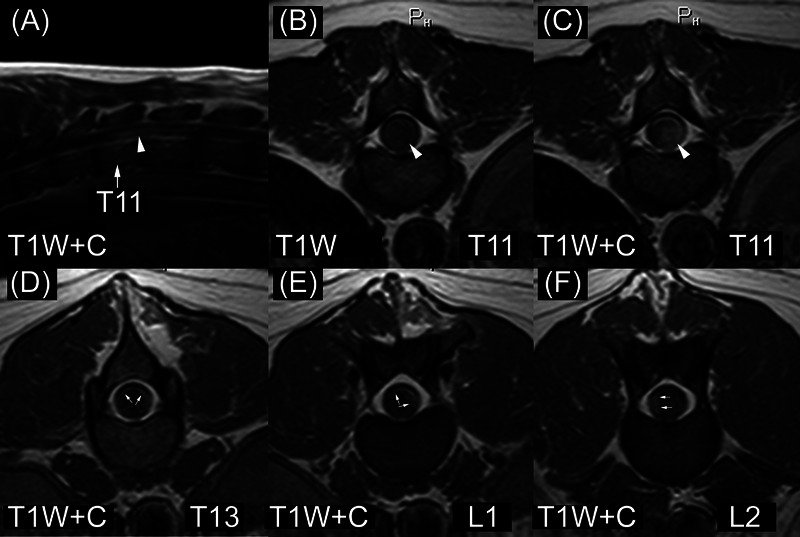
MRI of ependymoma in a 3‐year‐old Golden Retriever (field strength = 1 tesla). A–C, There is a contrast‐enhancing intramedullary lesion that is not centered on the central canal of the spinal cord (white arrowheads). D–F, There are multiple subtle regions of contrast enhancement involving the pia mater and white matter (small white arrows). These were thought to represent drop metastases. T11, eleventh thoracic vertebra, T1W, T1‐weighted image; T1W+C, T1‐weighted image made after gadolinium‐based contrast medium administration; T13, thirteenth thoracic vertebrae; L1, first lumbar vertebrae; L2, second lumbar vertebrae.

The third spinal cord ependymoma dog (Figure 6) was a 3‐year‐old, 27 kg, female spayed golden retriever (from WSU). She had a 14‐day history of paraparesis progressing to paraplegia. On MRI, she had numerous intramedullary lesions and multifocal meningeal contrast enhancement. The largest intramedullary lesion involved the eleventh and twelfth thoracic spinal cord segments. This lesion was not centered on the central canal of the spinal cord. Signal intensity was heterogeneous in T2W images. Lesion margins were not well‐circumscribed in both T1W and T2W images. The dog had syringohydromyelia cranial and caudal to the largest lesion. The dog had a smaller intramedullary lesion in the tenth thoracic spinal cord segment. This had similar signal intensity characteristics to the larger lesion. Furthermore, there were multiple plaque‐like regions of contrast enhancement in the periphery (pia mater and white matter) of the spinal cord caudal to the largest lesion. A biopsy was performed 1 day after the MRI. Treatments administered were not available.

## 4 DISCUSSION

The MRI features of canine ependymomas overlap with those of other types of neoplasia. Intracranial ependymomas consistently had forebrain (supratentorial) location, T1W and T2W signal heterogeneity, heterogeneous contrast enhancement, and high T2 signal intensity relative to NAGM. Like previous descriptions in textbooks, our cases had low or intermediate signal intensity in T1W images, high T2W signal intensity, variable contrast enhancement, and occasional intralesional cyst‐like structures and hemorrhage.^2^, ^4^ Margin delineation in our intracranial ependymomas was variable.

Intracranial ependymomas can mimic CPTs, which are the most common type of intraventricular neoplasia in dogs.^18^ The largest available case series of CPTs includes 32 dogs with MRI (9 papillomas and 23 carcinomas).^19^ In contrast to the ependymomas reported here, 10/32 of CPTs had an infratentorial location. Only 13 and 11 CPTs had signal intensity heterogeneity in T1W images and T2W images, respectively. Similar to our ependymomas, almost all CPTs (31/32) had contrast enhancement, but this was rated as heterogeneous in only 8 of 32 dogs. Intralesional cyst‐like structures were seen in 5 of 32 cases and apparent metastases in 8 of 32 cases. Intralesional cyst‐like structures and hemorrhage are seen in both CPTs and ependymomas.^2^, ^19^ In summary, supratentorial location, signal intensity heterogeneity (in both T1W and T2W images), and heterogeneous contrast enhancement may be used to prioritize an imaging diagnosis of ependymoma versus CPT. However, given its relatively high prevalence and variable appearance, the authors suggest that, in general, CPT should be considered the most likely diagnosis for any dog with an intraventricular mass. Less likely differentials should include ependymoma, oligodendroglioma, lymphoma, ventricular meningioma, and intraventricular neurocytoma.^4^, ^8^, ^9^, ^10^, ^12^ Both ventricular meningioma and intraventricular neurocytoma are rare. There is a paucity of literature on outcomes in dogs with histopathologically‐confirmed intraventricular neoplasia, therefore prognostic implications related to the type of neoplasia are largely unknown.^20^

The intra‐axial location of the ependymoma in the German shepherd dog was unexpected (Figure 2). The lesion was in the right olfactory region of the telencephalon. Interestingly, this location is common in human ependymomas. In a series of 49 cases of human supratentorial ependymoma, only 4 had an intraventricular location, and 44 had extraventricular parenchymal lesions extending from the pial surface to the ventricular margin.^21^ It is possible that this ependymoma arose from the olfactory recess of the lateral ventricle, which is often not visible in MRI.^22^ The authors suggest that ependymoma should be considered a rare cause of canine intra‐axial forebrain masses.

Another unexpected finding was the boxer with both an intraventricular ependymoma (right lateral ventricle) and an intra‐axial left occipital lobe mass. Unfortunately, histopathology of the intra‐axial mass was not available. It is probable that the mass was a glioma, as boxers are strongly predisposed.^23^ This mass did not contrast enhance, a finding typical of a low‐grade glioma.^24^ Other possible causes include a postictal change or, less likely, multifocal ependymoma.^25^ The latter is considered less likely because of the lack of contrast enhancement.

Intraventricular ependymomas have been described in a population of cats.^26^ Similar to the dogs reported here, feline ependymomas had a supratentorial location, T2W hyperintensity, variable contrast enhancement, and occasional intralesional cyst‐like structures and hemorrhage. Perilesional edema, obstructive hydrocephalus, and caudal transtentorial herniation were common. In contrast to our cases, two out of five feline ependymomas had T1W hyperintensity, and no cases had evidence of drop metastases. This study was limited to intraventricular masses and did not include intra‐axial or spinal cord masses.

Dogs in the spinal cord ependymoma group had contrast‐enhancing intramedullary lesions. Our findings are similar to previous reports of dogs with spinal cord ependymoma, glioma, and myelitis.^13^, ^27^, ^28^, ^29^, ^30^ We did not identify any specific features of canine spinal cord ependymoma. A “cap sign” has been reported in ∼45% of human spinal cord ependymoma cases and may help distinguish them from astrocytomas.^31^ The cap sign is defined as a rim of extremely low signal intensity on T2W sagittal plane images at the upper or lower pole of the tumor due to hemosiderin deposition from chronic hemorrhage.^31^ This sign has also been recently reported in one dog.^28^ None of the canine spinal cord ependymomas in the present study were positive for the cap sign. It is possible that this discrepancy reflects species‐related lesion differences, differences in spinal cord size relative to the spatial resolution of MRI, or an effect of a small sample size. The lack of T2*W images may also have prevented us from detecting low signals due to marginal chronic hemorrhagic changes. Canine spinal cord ependymomas had a variable relationship to the central canal of the spinal cord, as only one of three was centrally located. In contrast, approximately 90% of human spinal cord ependymomas are centrally located.^31^, ^32^

Evaluators were not asked to evaluate MRI studies for expansile enlargement of the vertebral canal caused by pressure atrophy associated with spinal cord ependymomas. That said, the images were evaluated after the fact by the first author. The first spinal cord ependymoma dog was positive for this finding (Figure 4), while the other two spinal cord ependymoma dogs were negative.

The golden retriever with multifocal canine spinal cord ependymoma (Figure 6) probably had drop metastases, which are defined as the dissemination of neoplasia via the cerebrospinal fluid. Drop metastases have been reported in dogs with CPTs and glioma and can be further classified based on their shape and location.^19^, ^33^, ^34^ This dog had diffuse leptomeningeal enhancement. These plaque‐like lesions could easily be overlooked or misinterpreted as evidence of meningomyelitis.

The age of the dogs reported herein is similar to previous reports. Our intracranial cases were 5, 11, and 10 years old, while our spinal cord cases were 7, 1, and 3 years old. The largest (nonimaging) case series of dogs with intracranial ependymoma included 5 cases of dogs from 3 to 11 years of age with a mean of 7.8.^3^ The largest case series of dogs with spinal cord ependymoma included 13 dogs with a mean age of 6.4 years.^13^ The majority of these dogs had thoracolumbar lesions (third thoracic through third lumbar spinal cord segments), which is also consistent with our findings. It is not surprising that two of our dogs were quite young, as there are several reports of spinal cord ependymoma in young dogs.^27^, ^28^, ^35^, ^36^

This study has a few limitations. First, despite conducting a broad search involving multiple institutions, case numbers are low. This is a reflection of the rarity of ependymoma in dogs and the difficulty distinguishing it from other types of neoplasia using histopathology and immunohistochemistry.^3^ Because of the low case numbers reported herein, caution should be used in extrapolating our results to the clinic. Second, specific technical parameters used for MRI studies were not recorded. It is unknown how these technical parameters could have influenced imaging characteristics. Furthermore, in dogs with spinal cord ependymoma, brain imaging was not performed to screen for a primary tumor that may have drop metastasized. Finally, this study was not designed to investigate accuracy, reliability, or factors associated with outcomes.

In conclusion, the canine intracranial and spinal cord ependymomas in our study had MRI features that overlap those seen in other diseases of the brain and spinal cord. Although intraventricular masses and association with the central canal are common presentations of canine ependymoma, our study shows that ependymomas can also form intra‐axial or intramedullary masses that are not clearly associated with the ventricles or central canal. The authors hope our results are helpful in creating prioritized differential diagnosis lists. Image evaluators should scrutinize images to rule out drop metastases. A biopsy is recommended to confirm the diagnosis

## LIST OF AUTHOR CONTRIBUTIONS

### Category 1

(a) Conception and design: Griffin, Stevenson, Hecht, Porter, Boudreau, Mai.
(b) Acquisition of data: Stevenson, Nelson, Chen, Hecht, Porter, Boudreau, Specchi, Bernardini, Mai
(c) Analysis and interpretation of data: Griffin, Stevenson, Hecht, Specchi, Mai

### Category 2

(a) Drafting the article: Griffin, Stevenson
(b) Revising article for intellectual content: Griffin, Stevenson, Nelson, Chen, Hecht, Porter, Boudreau, Specchi, Bernardini, Mai

### Category 3

(a) Final approval of the completed article: Griffin, Stevenson, Nelson, Chen, Hecht, Porter, Boudreau, Specchi, Bernardini, Mai

## CONFLICT OF INTEREST STATEMENT

The authors declare no conflict of interest.

## DATA ACCESSIBILITY STATEMENT

The data used in the study are available from the corresponding author upon reasonable request.

## PREVIOUS PRESENTATION OR PUBLICATION DISCLOSURE

NA

## REPORTING CHECKLIST DISCLOSURE

The EQUATOR network checklist was not used.

## Supporting information



Supporting information
