# Supplementary material for: MRI findings in six dogs with ependymoma of the brain and spinal cord
Source: Vet Radiol Ultrasound. 2024 Dec 16;66(1):e13477. doi: 10.1111/vru.13477 (PMC11649882; doi:10.1111/vru.13477)
Supplement: Supplementary file 1 — Supporting information [file VRU-66-0-s001.docx]

| **Supplementary Table 1. MRI Parameters for Intracranial Ependymoma Dogs** | | | | | | | | |
| --- | --- | --- | --- | --- | --- | --- | --- | --- |
|  | Sequence Name | TR (ms) | TE (ms) | FOV (mm) | Matrix | Slice Thk (mm) | Gap (%) | Notes |
| Intracranial Dog 1, Siemens Magnetom Verio, 3 tesla, Malvern, PA, USA | | | | | | | | |
|  | 2D T2w Sag TSE | 3700 | 86 | 250 | 320 X 240 | ≤ 3 | 10 | FS |
|  | 2D T2w Trans TSE | 4150 | 99 | 150 | 256 X 212 | 4 | 10 |  |
|  | 3D T1w IR-GRE^ab^ | 2100 | 2.9 | 200 | 320 X 320 | 0.6 | 0 | FS  TI = 900 ms |
|  | 2D T2*w Trans GRE | 730 | 19.9 | 150 | 192 X 163 | 4 | 10 | FS |
|  | 2D T2w FLAIR Trans TSE | 7000 | 94 | 150 | 256 X 228 | 4 | 10 | TI = 2215 ms |
| Intracranial Dog 2, Siemens Magnetom Symphony, 1.5 tesla, Malvern, PA, USA | | | | | | | | |
|  | 2D T2w Sag TSE | 4820 | 115 | 200 | 256 X 230 | 3 | 10 | FS |
|  | 2D T2w Trans TSE | 4090 | 91 | 160 | 256 X 205 | 4 | 0 |  |
|  | 2D T1w Trans TSE^b^ | 523 | 12 | 160 | 256 X 192 | 4 | 0 | FS |
|  | 2D T2*w Trans GRE | 1020 | 26 | 160 | 256 X 192 | 4 | 0 | FS |
|  | 2D T2w FLAIR Trans TSE | 9000 | 78 | 160 | 256 X 192 | 4 | 0 | TI = 2500 ms |
| Intracranial Dog 3, Paramed MrJ, 0.22 tesla, Genoa, IT | | | | | | | | |
|  | 2D T2w Sag FSE | 3200 | 120 | 200 | 320 X 192 | 4.5 | 10 |  |
|  | 2D T2w Trans FSE | 4155 | 120 | 200 | 320 X 192 | 5 | 10 |  |
|  | 2D T1w Trans FSE^b^ | 730 | 24 | 200 | 320 X 192 | 5 | 10 |  |
|  | 2D T2w Turbo FLAIR Dors | 4633 | 90 | 200 | 320 X 192 | 5 | 10 | TI = 1150 ms |
|  | | | | | | | | |
| Abbreviations: TR- time of repetition; ms- milliseconds; TE- time of echo; FOV- field-of-view; Slice thk- slice thickness; Gap- interslice gap; 2D- two dimensional; T2w- T2-weighted; Sag- sagittal; TSE- turbo spin echo; FS- fat saturation; Trans- transvers; 3D- three dimensional; T1w- T1-weighted, IR- inversion recovery; GRE- gradient recalled echo; TI- time of inversion; T2*w- T2*-weighted, FLAIR- fluid attenuated inversion recovery, FSE- fast spin echo; Dors- dorsal | | | | | | | | |
| ^a^magnetization prepared rapid gradient-echo (MPRAGE), reconstructed in 3 planes  ^b^repeated before and after contrast medium injection | | | | | | | | |

| **Supplementary Table 2. MRI Parameters for Spinal Cord Ependymoma Dogs** | | | | | | | | |
| --- | --- | --- | --- | --- | --- | --- | --- | --- |
|  | Sequence Name | TR (ms) | TE (ms) | FOV (mm) | Matrix | Slice Thk (mm) | Gap (%) | Notes |
| Spinal Cord Dog 1, Paramed MrJ, 0.22 tesla, Genoa, IT | | | | | | | | |
|  | 2D T2w Sag FSE | 3100 | 120 | 200 | 256 X 192 | 3.5 | 10 |  |
|  | 2D T2w Trans FSE | 3910 | 120 | 200 | 256 X 192 | 3.5 | 10 |  |
|  | 2D T1w Sag FSE^a^ | 521 | 26 | 200 | 304 X 192 | 3.5 | 10 |  |
|  | 2D T1w Trans FSE^a^ | 741 | 26 | 200 | 304 X 192 | 3.5 | 10 |  |
| Spinal Cord Dog 2, Philips Gyroscan, 1 tesla, Andover, MA, USA | | | | | | | | |
|  | 2D T2w Sag TSE | 3500 | 120 | 314 | 512 X 512 | 3 | 10 |  |
|  | 2D T2w Trans TSE | 5052 | 100 | 160 | 512 X 512 | 3.5 | 10 |  |
|  | 2D T1w Trans TSE | 674 | 15 | 160 | 512 X 512 | 3.5 | 10 | Pre-contrast |
|  | 2D T1w Trans TSE | 594 | 13 | 160 | 512 X 512 | 3.5 | 10 | Post-contrast |
| Spinal Cord Dog 3, Philips Gyroscan, 1 tesla, Andover, MA, USA | | | | | | | | |
|  | 2D T2w Sag TSE | 3500 | 120 | 384 | 512 X 512 | 3 | 17 |  |
|  | 2D T2w Trans TSE | 5064 | 100 | 160 | 512 X 512 | 3.5 | 17 |  |
|  | 2D T1w Trans TSE^a^ | 674 | 15 | 160 | 512 X 512 | 3.5 | 14 |  |
|  | | | | | | | | |
| Abbreviations: TR- time of repetition; ms- milliseconds; TE- time of echo; FOV- field-of-view; Slice thk- slice thickness; Gap- interslice gap; 2D- two dimensional; T2w- T2-weighted; Sag- sagittal; FSE- fast spin echo; Trans- transverse; TSE- turbo spin echo; T1w- T1-weighted | | | | | | | | |
| ^a^repeated before and after contrast medium injection | | | | | | | | |
